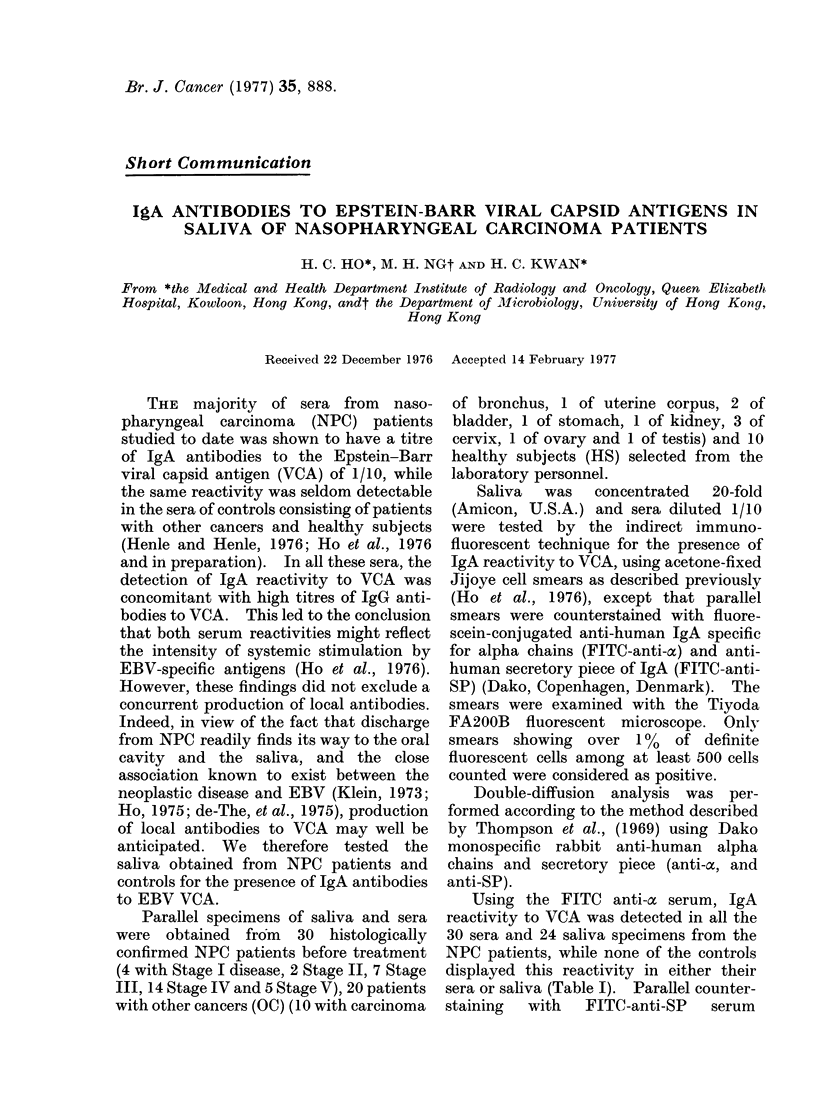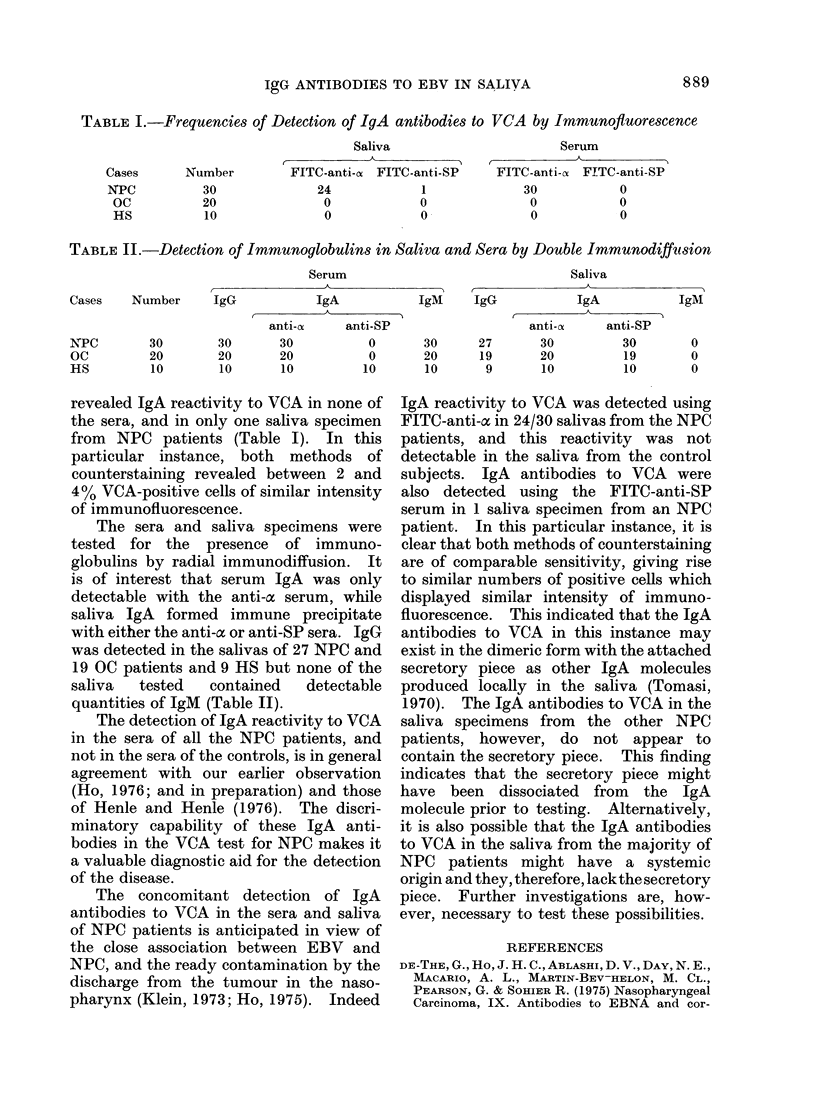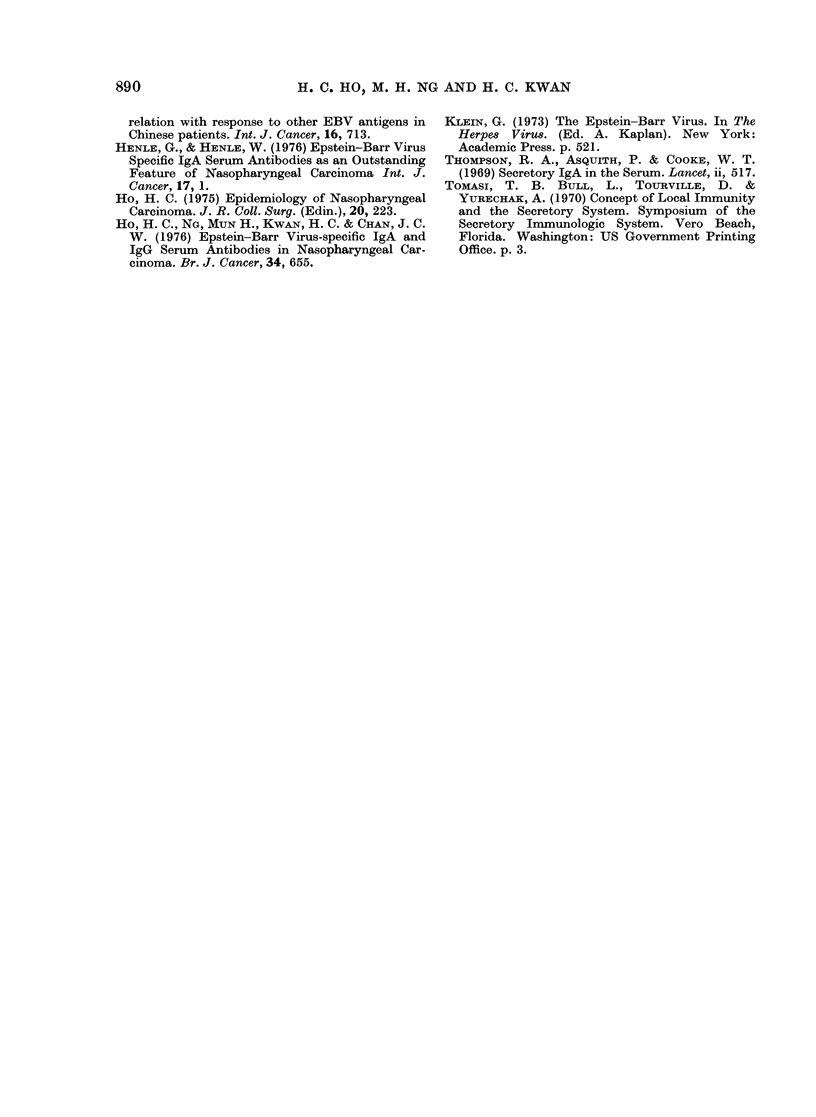# IgA antibodies to Epstein-Barr viral capsid antigens in saliva of nasopharyngeal carcinoma patients.

**DOI:** 10.1038/bjc.1977.134

**Published:** 1977-06

**Authors:** H. C. Ho, M. H. Ng, H. C. Kwan


*Br. J. Cancer* (1977) **35**, 888.

### *Short Communication*

### IgA ANTIBODIES TO EPSTEIN-BARR VIRAL CAPSID ANTIGENS IN SALIVA OF NASOPHARYNGEAL CARCINOMA PATIENTS

H. C. HO*, M. H. NG† AND H. C. KWAN*

*From *the Medical and Health Department Institute of Radiology and Oncology, Queen Elizabeth Hospital, Kowloon, Hong Kong, and† the Department of Microbiology, University of Hong Kong, Hong Kong*

Received 22 December 1976 Accepted 14 February 1977

THE majority of sera from nasopharyngeal carcinoma (NPC) patients studied to date was shown to have a titre of IgA antibodies to the Epstein–Barr viral capsid antigen (VCA) of 1/10, while the same reactivity was seldom detectable in the sera of controls consisting of patients with other cancers and healthy subjects (Henle and Henle, 1976; Ho *et al.*, 1976 and in preparation). In all these sera, the detection of IgA reactivity to VCA was concomitant with high titres of IgG antibodies to VCA. This led to the conclusion that both serum reactivities might reflect the intensity of systemic stimulation by EBV-specific antigens (Ho *et al.*, 1976). However, these findings did not exclude a concurrent production of local antibodies. Indeed, in view of the fact that discharge from NPC readily finds its way to the oral cavity and the saliva, and the close association known to exist between the neoplastic disease and EBV (Klein, 1973; Ho, 1975; de-The, *et al.*, 1975), production of local antibodies to VCA may well be anticipated. We therefore tested the saliva obtained from NPC patients and controls for the presence of IgA antibodies to EBV VCA.

Parallel specimens of saliva and sera were obtained from 30 histologically confirmed NPC patients before treatment (4 with Stage I disease, 2 Stage II, 7 Stage III, 14 Stage IV and 5 Stage V), 20 patients with other cancers (OC) (10 with carcinoma of bronchus, 1 of uterine corpus, 2 of bladder, 1 of stomach, 1 of kidney, 3 of cervix, 1 of ovary and 1 of testis) and 10 healthy subjects (HS) selected from the laboratory personnel.

Saliva was concentrated 20-fold (Amicon, U.S.A.) and sera diluted 1/10 were tested by the indirect immunofluorescent technique for the presence of IgA reactivity to VCA, using acetone-fixed Jijoye cell smears as described previously (Ho *et al.*, 1976), except that parallel smears were counterstained with fluorescein-conjugated anti-human IgA specific for alpha chains (FITC-anti-$\alpha$) and anti-human secretory piece of IgA (FITC-anti-SP) (Dako, Copenhagen, Denmark). The smears were examined with the Tiyoda FA200B fluorescent microscope. Only smears showing over 1% of definite fluorescent cells among at least 500 cells counted were considered as positive.

Double-diffusion analysis was performed according to the method described by Thompson *et al.*, (1969) using Dako monospecific rabbit anti-human alpha chains and secretory piece (anti-$\alpha$, and anti-SP).

Using the FITC anti-$\alpha$ serum, IgA reactivity to VCA was detected in all the 30 sera and 24 saliva specimens from the NPC patients, while none of the controls displayed this reactivity in either their sera or saliva (Table I). Parallel counter-staining with FITC-anti-SP serum

TABLE I.—*Frequencies of Detection of IgA antibodies to VCA by Immunofluorescence*

| Cases | Number | Saliva | | Serum | |
|---|---|---|---|---|---|
| | | FITC-anti-α | FITC-anti-SP | FITC-anti-α | FITC-anti-SP |
| NPC | 30 | 24 | 1 | 30 | 0 |
| OC | 20 | 0 | 0 | 0 | 0 |
| HS | 10 | 0 | 0 | 0 | 0 |

TABLE II.—*Detection of Immunoglobulins in Saliva and Sera by Double Immunodiffusion*

| Cases | Number | Serum | | | | Saliva | | | |
|---|---|---|---|---|---|---|---|---|---|
| | | IgG | IgA | | IgM | IgG | IgA | | IgM |
| | | | anti-α | anti-SP | | | anti-α | anti-SP | |
| NPC | 30 | 30 | 30 | 0 | 30 | 27 | 30 | 30 | 0 |
| OC | 20 | 20 | 20 | 0 | 20 | 19 | 20 | 19 | 0 |
| HS | 10 | 10 | 10 | 10 | 10 | 9 | 10 | 10 | 0 |

revealed IgA reactivity to VCA in none of the sera, and in only one saliva specimen from NPC patients (Table I). In this particular instance, both methods of counterstaining revealed between 2 and 4% VCA-positive cells of similar intensity of immunofluorescence.

The sera and saliva specimens were tested for the presence of immunoglobulins by radial immunodiffusion. It is of interest that serum IgA was only detectable with the anti-α serum, while saliva IgA formed immune precipitate with either the anti-α or anti-SP sera. IgG was detected in the salivas of 27 NPC and 19 OC patients and 9 HS but none of the saliva tested contained detectable quantities of IgM (Table II).

The detection of IgA reactivity to VCA in the sera of all the NPC patients, and not in the sera of the controls, is in general agreement with our earlier observation (Ho, 1976; and in preparation) and those of Henle and Henle (1976). The discriminatory capability of these IgA antibodies in the VCA test for NPC makes it a valuable diagnostic aid for the detection of the disease.

The concomitant detection of IgA antibodies to VCA in the sera and saliva of NPC patients is anticipated in view of the close association between EBV and NPC, and the ready contamination by the discharge from the tumour in the nasopharynx (Klein, 1973; Ho, 1975). Indeed IgA reactivity to VCA was detected using FITC-anti-α in 24/30 salivas from the NPC patients, and this reactivity was not detectable in the saliva from the control subjects. IgA antibodies to VCA were also detected using the FITC-anti-SP serum in 1 saliva specimen from an NPC patient. In this particular instance, it is clear that both methods of counterstaining are of comparable sensitivity, giving rise to similar numbers of positive cells which displayed similar intensity of immunofluorescence. This indicated that the IgA antibodies to VCA in this instance may exist in the dimeric form with the attached secretory piece as other IgA molecules produced locally in the saliva (Tomasi, 1970). The IgA antibodies to VCA in the saliva specimens from the other NPC patients, however, do not appear to contain the secretory piece. This finding indicates that the secretory piece might have been dissociated from the IgA molecule prior to testing. Alternatively, it is also possible that the IgA antibodies to VCA in the saliva from the majority of NPC patients might have a systemic origin and they, therefore, lack the secretory piece. Further investigations are, however, necessary to test these possibilities.

### REFERENCES

DE-THE, G., HO, J. H. C., ABLASHI, D. V., DAY, N. E., MACARIO, A. L., MARTIN-BEV-HELON, M. CL., PEARSON, G. & SOHIER R. (1975) Nasopharyngeal Carcinoma, IX. Antibodies to EBNA and correlation with response to other EBV antigens in Chinese patients. *Int. J. Cancer*, **16**, 713.

HENLE, G., & HENLE, W. (1976) Epstein–Barr Virus Specific IgA Serum Antibodies as an Outstanding Feature of Nasopharyngeal Carcinoma *Int. J. Cancer*, **17**, 1.

HO, H. C. (1975) Epidemiology of Nasopharyngeal Carcinoma. *J. R. Coll. Surg.* (Edin.), **20**, 223.

HO, H. C., NG, MUN H., KWAN, H. C. & CHAN, J. C. W. (1976) Epstein–Barr Virus-specific IgA and IgG Serum Antibodies in Nasopharyngeal Carcinoma. *Br. J. Cancer*, **34**, 655.

KLEIN, G. (1973) The Epstein–Barr Virus. In *The Herpes Virus*. (Ed. A. Kaplan). New York: Academic Press. p. 521.

THOMPSON, R. A., ASQUITH, P. & COOKE, W. T. (1969) Secretory IgA in the Serum. *Lancet*, ii, 517.

TOMASI, T. B. BULL, L., TOURVILLE, D. & YURECHAK, A. (1970) Concept of Local Immunity and the Secretory System. Symposium of the Secretory Immunologic System. Vero Beach, Florida. Washington: US Government Printing Office. p. 3.